# A metabotropic glutamate receptor affects the growth and development of *Schistosoma japonicum*

**DOI:** 10.3389/fmicb.2022.1045490

**Published:** 2022-11-30

**Authors:** Xiaoling Wang, Shaoyun Cheng, Xiangyu Chen, Wei Zhang, Yuxiang Xie, Wanling Liu, Yanmin You, Cun Yi, Bingkuan Zhu, Mengjie Gu, Bin Xu, Yan Lu, Jipeng Wang, Wei Hu

**Affiliations:** ^1^National Institute of Parasitic Diseases, Chinese Center for Disease Control and Prevention (Chinese Center for Tropical Diseases Research), NHC Key Laboratory of Parasite and Vector Biology, WHO Collaborating Center for Tropical Diseases, National Center for International Research on Tropical Diseases, Shanghai, China; ^2^State Key Laboratory of Genetic Engineering, Ministry of Education Key Laboratory of Contemporary Anthropology, Department of Microbiology and Microbial Engineering, School of Life Sciences, Fudan University, Shanghai, China; ^3^Department of Infectious Diseases, Huashan Hospital, Fudan University, Shanghai, China; ^4^College of Life Sciences, Inner Mongolia University, Hohhot, China

**Keywords:** *Schistosoma japonicum*, metabotropic glutamate receptor, development, double-stranded RNA, liver fibrosis

## Abstract

Schistosomiasis is a zoonotic parasitic disease caused by schistosome infection that severely threatens human health. Therapy relies mainly on single drug treatment with praziquantel. Therefore, there is an urgent need to develop alternative medicines. The glutamate neurotransmitter in helminths is involved in many physiological functions by interacting with various cell-surface receptors. However, the roles and detailed regulatory mechanisms of the metabotropic glutamate receptor (mGluR) in the growth and development of *Schistosoma japonicum* remain poorly understood. In this study, we identified two putative mGluRs in *S. japonicum* and named them *Sj*GRM7 (Sjc_001309, similar to GRM7) and *Sj*GRM (Sjc_001163, similar to mGluR). Further validation using a calcium mobilization assay showed that *Sj*GRM7 and *Sj*GRM are glutamate-specific. The results of *in situ* hybridization showed that *Sj*GRM is mainly located in the nerves of both males and gonads of females, and *Sj*GRM7 is principally found in the nerves and gonads of males and females. In a RNA interference experiment, the results showed that *Sj*GRM7 knockdown by double-stranded RNA (dsRNA) in *S. japonicum* caused edema, chassis detachment, and separation of paired worms *in vitro*. Furthermore, dsRNA interference of *Sj*GRM7 could significantly affect the development and egg production of male and female worms *in vivo* and alleviate the host liver granulomas and fibrosis. Finally, we examined the molecular mechanisms underlying the regulatory function of mGluR using RNA sequencing. The data suggest that *Sj*GRM7 propagates its signals through the G protein-coupled receptor signaling pathway to promote nervous system development in *S. japonicum*. In conclusion, *Sj*GRM7 is a potential target for anti-schistosomiasis. This study enables future research on the mechanisms of action of *Schistosomiasis japonica* drugs.

## Introduction

Schistosomiasis is a zoonotic parasitic disease affecting approximately 290 million people worldwide (Afshin et al., 2019). Schistosomiasis mainly affects humans via *Schistosoma mansoni*, *Schistosoma haematobium*, and *Schistosoma japonicum*. *S japonicum* is localized to Asia, primarily the Philippines and China (Colley et al., 2014). These parasites require two hosts to complete their life cycle, including freshwater snails (intermediate host) for asexual reproduction and mammals (final host) for sexual reproduction (Talla et al., 1990).

Currently, praziquantel is the optimal drug to treat schistosomiasis (Zdesenko and Mutapi, 2020). There is an urgent need to develop new therapeutic agents because of the risk of drug resistance due to long-term single-agent use. The nervous system of schistosomes controls neuromuscular signaling related to movement, host attachment and migration, as well as sensory neurons located at the surface that may be involved in host–parasite interactions. If the neuronal connection is interrupted, parasites may be eliminated from the hosts. Therefore, the nervous system of *Schistosoma* is a promising target for therapeutic drug development.

L-Glutamate is a major neurotransmitter in both vertebrates and invertebrates. Glutamate neuronal signaling has been detected in *Caenorhabditis elegans* (Bono and Villu Maricq, 2005), *Fasciola hepatica* (Brownlee and Fairweather, 1996), *Hymenolepis diminuta* (Webb and Eklove, 1989), and *S. mansoni* (Taman and Ribeiro, 2011) and can interact with various cell surface receptors for signal transduction, including ionotropic gated channels and metabotropic glutamate receptors (mGluRs, also known as GRMs). mGluRs are structurally related to γ-aminobutyric acid B receptor (GABABR), calcineurin, and other receptors in the G protein-coupled receptor (GPCR) family (Pin et al., 2003). GPCRs are the largest family of membrane proteins for cellular communication in living organisms. These receptors can detect signal molecules in the extracellular environment, such as ions, hormones, light, neurotransmitters, amino acids, and peptides, and then trigger a series of intracellular signal transduction pathways to generate the corresponding physiological effects (Weis and Kobilka, 2018). Based on sequence homology and similarity, GPCRs are classified into four prominent families: class A (rhodopsin-like), class B (secretin-like), class C (metabotropic glutamate receptor-like), class F (frizzled/smoothened like), and others. Classes A, B, and C are the main receptor families (Bockaert et al., 2002). C-family GPCRs have a unique modular structure consisting of an N-terminal extracellular domain (ECD), a C-terminal intracellular domain, and a 7-Transmembrane (7-TM) fragment. The ECD carries a conserved Venus Flytrap module (VFT) containing a glutamate-binding site and is linked to the 7-TM region by a short cysteine-rich linker (Ferraguti and Shigemoto, 2006; Niswender and Conn, 2010). They are divided into three categories according to their sequence similarity, pharmacological properties, and signal transduction mechanisms (Pin et al., 2003). Gq/11 protein coupling and signaling by the intracellular calcium and inositol phospholipid pathway changes in group I (mGluR1 and mGluR5). The group II (mGluR2 and mGluR3) and group III (mGluR4, mGluR6, mGluR7, and mGluR8) classes bind to Gi/o proteins primarily via inhibition of adenosine acid cyclase, which in turn reduces intracellular cAMP signaling (Pin et al., 2003).

In this study, we first obtained two mGluRs in *S. japonicum* using bioinformatics and then investigated their functions *in vitro* and *in vivo*. The results showed that *Sj*GRM7 is extremely important for the normal physiological activity, growth, development, and egg production of *S. japonicum*. Finally, we used RNA sequencing (RNA-seq) to preliminarily explore the regulatory role of *Sj*GRM7 on downstream signaling pathways, prompting further drug development research to treat *S. japonicum*.

## Materials and methods

### Sequence analysis

#### Identification, phylogenetic analysis, and multiple sequence alignment of *Schistosoma japonicum* metabotropic glutamate receptors

*Schistosoma japonicum* mGluRs were identified by combining a hidden Markov model (HMM) and a protein basic local alignment search tool (blastp). The reviewed mGluRs from UniProt were used to generate HMM models, which were subsequently used for HMM searches of *S. japonicum* protein sequences. Blastp was also incorporated using *S. mansoni* mGluRs against the *S. japonicum* protein sequences. These two results were combined, followed by a TM domain prediction. Proteins with more than three TM domains were considered mGluRs, including *Sj*c_0001163 and *Sj*c_0001309.

A phylogenetic tree of mGluR was constructed using the maximum-likelihood approach. Sequences of *S. mansoni* and non-flatworm species were obtained from Tulio et al. (2014) and Ramos-Vicente et al. (2018). Sequences from *Schmidtea mediterranea* were obtained using Blastp (Grohme et al., 2018). MUSCLE in MEGAX was used for the alignment. Maximum likelihood analysis was conducted using RAxML with the WAG + I + G + F and 1,000 iterations of ultrafast bootstrapping. The tree was illustrated using the iTOL software.

Representative sequences in the phylogenetic tree were chosen for multiple alignments of the mGluR residues involved in ligand binding. The residue numbers at the top of the graph correspond to the human mGluR1. The figure was prepared using JalView v2.10.4b1.

#### Protein structure and binding pocket prediction

The structures of the two mGluRs in *S. japonicum* were determined using the ColabFold software. The position of the ligand (Protein Data Bank entries: GLU) was predicted using Dock Prep and AutoDock Vina. The *Homo sapiens* mGluR2 (Protein Data Bank identifier: 7MTR) was exhibited with Chimera.

### Cell culture, transfection, and calcium mobilization assay

To improve the expression of the two mGluRs in *S. japonicum* within mammalian cells, the Kozak GCC ACC sequence was added before the start codon of the forward primer. PCR amplified the full length of the modified *Sj*GRM and *Sj*GRM7, and the amplified cDNA was cloned into the prK5 expression vector. The restriction endonuclease site of prK5 was located between *Mlu*I and *Sal*I, and the constructed plasmid was verified by DNA sequencing. Human embryonic kidney 293T cells (HEK293T) were transfected with two mGluRs and a heterotrimeric G-protein, Gα16, using Lipofectamine 2000 (Invitrogen), and incubated at 37°C with 5% CO_2_ for 24 h. Subsequently, the medium was discarded and 1 mM Fluo-4 AM fluorescent dye (Invitrogen) was added and incubated for 1 h (Caers et al., 2014). Next, different concentrations of L-glutamate, L-aspartate, glycine, GABA, and glutamine (glutamate derivative) (Sangon Bioengineering Co., Ltd., Shanghai, China) were added. A FlexStation instrument was used to excite the dye using a 485 nm laser. The fluorescence was recorded at 525 nm to detect the calcium mobilization signal in living cells and determine the immediate response after stimulation.

### Experimental animals and *Schistosoma japonicum*

Female Kunming mice of specific pathogen-free (SPF) grade, 6 weeks old, and weighing 18 ± 2 g, were purchased from Slack Laboratory Animal Co., Ltd. (Shanghai, China) and raised in the SPF animal room of the Institute of Parasitic Diseases, Chinese Center for Disease Control and Prevention. Cercariae of *S. japonicum* (mainland China strain) were provided by the vector room of the Institute of Parasitic Diseases, Chinese Center for Disease Control and Prevention.

### Preparation and collection of *Schistosoma japonicum* at different developmental stages

Eight Kunming mice were randomly divided into two groups, the *Sj*GRM7 double-stranded RNA (dsRNA) group and the GFP control group. Each mouse was infected with 200 ± 10 cercariae via abdominal skin. Kunming mice were euthanized and dissected at 14, 16, 18, 20, 22, 24, 26, 28, and 30 days post infection (dpi). The worms were harvested, and the male and female worms were separated with a soft brush after washing with liquid nitrogen and stored in a −80°C freezer (Wang et al., 2017). *S. japonicum* at 26 to 30 dpi were killed with 0.6 M magnesium chloride, followed by 4% paraformaldehyde (PFA) in phosphate buffered saline with 0.3% Tween20 (PBSTx), incubated for 4 h at room temperature (RT), sequentially dehydrated in 50% methanol in PBSTx, and finally stored at −20°C.

### *In situ* hybridization

Before hybridization, the 10X DIG RNA Labeling Kit (Roche, Germany) was used to synthesize digoxigenin-labeled RNA probes. Primer sequences used are listed in Supplementary Table 6. The worms were removed from −20°C and rehydrated in 50% methanol and PBSTx for 5 min, and a bleaching solution was added for 1 h (Cogswell et al., 2011). Worms were rinsed with PBSTx for 5 min, and 5 μg/ml proteinase K (Invitrogen) was added to PBSTx for 45 min and fixed with 4% PFA in PBSTx for 10 min at RT. The worms were then placed in a prehybe solution (50% deionized formamide, 5× SSC, 1 mg/ml torula yeast RNA, 1% Tween20) at 52°C for 2 h. Hybridization was carried out for 16 h at 52°C in the hybe solution [10% dextran sulfate (Sangon) in prehybe solution] and riboprobe (200 ng/ml; Sigma). The hybe solution was removed and washed in wash hybe, 2xSSC, and 0.2xSSC for 30 min (2 times) at 52°C and washed with 0.1 M Tris pH 7.5, 0.15 M NaCl, 0.1% Tween-20 (TNT) for 10 min at RT. Then, fluorescence *in situ* hybridization (FISH) block solution (5% horse serum, 0.5% Roche Western Blocking Reagent in TNT) was added for 2 h at RT, and anti-DIG-POD (1:1000) was replaced in the FISH block solution, and the solution was incubated overnight at 4°C. The reaction was washed for 5, 10, and 20 min six times with TNT. The cells were incubated in a tyramide solution for 10 min and washed for 5 min with TNT, and incubated with 1 μg/ml 4′,6-diamidino-2-phenylindole (DAPI) 4 h at RT. The cells were observed in 80% glycerol in PBS and imaged using a Nikon A1 upright laser scanning confocal microscope.

### Synthesis of double-stranded RNA

Primers were designed, and the primer sequences are listed in Supplementary Table 6. Polymerase chain reaction (PCR) was then performed. DNA sequencing confirmed that the product was extracted and recovered from agarose gels and its identity was confirmed by Tsingke Biotechnology Co., Ltd. The recovered product was prepared and purified using a MEGAscript TM T7 High Yield Transcription Kit (Thermo). The size and integrity of the dsRNA were verified using 1% agarose gel electrophoresis. Finally, the correctly synthesized dsRNA was aliquoted and stored at −80°C.

### Quantitative polymerase chain reaction

TRIzol reagent (Takara, Japan) was used to extract total RNA from *S. japonicum* and mouse livers, and reverse transcription was performed using a cDNA reverse transcription kit (Takara, Japan). The cDNA was used as a template for quantitative polymerase chain reaction (qPCR) with the SYBR Green Fast qPCR Master Mix (Takara, Japan) and 0.2 μM forward and reverse primers. The amplification conditions were 94°C for 5 min, followed by 38 cycles of 94°C for 30 s, 55°C for 30 s, 72°C for 50 s, and 72°C for 10 min. Primer sequences are listed in Supplementary Table 6. *S. japonicum* qPCR data were normalized relative to the endogenous gene 26S proteasome non-ATPase (PSMD), which served as an internal control. Gene expression was calculated using the 1000 × 2^–△^ *^CT^* method, as described previously (Li et al., 2018). The relative expression of α-smooth muscle actin (SMA), collagen I, and collagen 3 in the mouse liver was calculated using the 1000 × 2^–△^^△^ *^Ct^* method and glyceraldehyde-3-phosphate dehydrogenase (GAPDH) as an endogenous control to normalize mRNA levels (Hu et al., 2020).

### RNA interference of *Schistosoma japonicum in vitro* and sample collection

Adult paired *S. japonicum* (30 dpi) were obtained and cultured in Dulbecco’s modified Eagle medium (DMEM; Gibco) containing 10% fetal bovine serum (FBS; Gibco). dsRNA was added on days 1, 3, and 5 of the *in vitro* culture and the phenotype of the worms was observed on day 7. After sample collection, the corresponding gene changes were evaluated from different samples using qPCR, and RNA-seq was performed to explore the downstream signaling pathways. Three independent biological replicates were used in this experiment. Each group consisted of three replicate wells for each biological experiment, with six pairs of adult worms in each replicate well and six worms for qPCR as well as RNA-seq.

### RNA interference of *Schistosoma japonicum in vivo* and sample collection

Each mouse was infected with 60 cercariae via abdominal skin penetration and randomly divided into two groups, with four mice per group. *Sj*GRM7 and green fluorescent protein (GFP) dsRNA were injected into the tail vein for interference at 1, 6, 10, 14, 18, 22, and 26 dpi, and the influence of *S. japonicum* on the growth and development of the mice was observed by harvesting worms at 30 dpi (Li et al., 2018). For the egg-laying model, eight mice were randomly divided into two groups, the *Sj*GRM7 dsRNA group and the GFP control group, 40 cercariae were used per mouse. Ten micrograms of dsRNA were injected into the tail vein at 26, 30, 34, and 38 dpi to achieve long-term continuous interference with *Sj*GRM7. Mice were euthanized at 42 days and the liver was collected to detect changes in the number of hepatic eggs and the liver pathology observed (see the section “Histological evaluation”). Finally, five male and five female *S. japonicum* individuals were separated. A small lobe of the liver was washed with diethyl pyrocarbonate (DEPC)/PBS and placed in liquid nitrogen for quick freezing for mRNA quantification. The remaining worms were fixed in 95% alcohol, 3% formaldehyde, and 2% glacial acetic acid (AFA).

### RNA sequencing data

Total RNA extraction, library construction, and RNA-seq were conducted by the Novagene Technology Corporation (Beijing, China). RNA was extracted from the worms using a modified phenol-chloroform method and column purification. The integrity and quality of the total RNA were examined using a Nanodrop ND-2000 spectrophotometer (Thermo, United States) and an Agilent 2100 Bioanalyzer (Agilent, United States). Pooled RNA was used to construct a library using an Illumina TruSeq RNA Sample Prep Kit (Illumina, San Diego, CA, United States) with a Ribo-Zero Magnetic Kit for RNA depletion, according to the TruSeq RNA Sample Preparation Guide. This library was subsequently sequenced on the Illumina Nova 6000 platform to obtain 150 bp paired-end (PE) reads. The sequencing data were deposited in the NCBI Sequence Read Archive (SRA) database under accession numbers (PRJNA880502).

Quality control (QC) of raw sequencing data was performed using the FASTQC program (Andrews, 2013). Low-quality reads and adapter sequences were trimmed using FASTP v.0.20.1. (Parameters: -q 15 -u 40 -n 5 -l 15) (Chen et al., 2018). The clean reads were mapped to the chromosome-level *S. japonicum* reference genome (*Sj*V3) using HISAT2 v2.1.0 (Kim et al., 2019) with default parameters. Gene-level assignments were then performed to estimate transcript abundance using featureCounts 1.6.4 (Liao et al., 2013). Principal component analysis (PCA) was performed using the prcomp function in the stats (v3.6.0) R package. Hierarchical clustering analysis (HCA) was performed using Pheatmap^1^. The R package DEseq2 v1.26.0 (Love et al., 2014) was used to perform differential expression analysis. Gene Ontology (GO) enrichment analysis was performed using the R package clusterProfiler (Yu et al., 2012). The *p*-values were corrected for multiple hypothesis testing using the Benjamini–Hochberg false discovery rate procedure (adjusted *p*-value).

### Observation of the effect on the growth and development of *Schistosoma japonicum* after dsRNA interference *in vivo*

The AFA-fixed body was photographed under a microscope and its length was measured using ImageJ^2^. We measured worm length using all schistosomes from each mouse, excluding the six utilized for qPCR. AFA absorption, followed by the addition of deionized water, glacial acetic acid, 35% ethanol, 50% ethanol, and 70% ethanol for decolorization, was carried out for carmine staining and liquid separation. Separation liquid was discarded for 35%, 50%, and 70% ethanol dehydration. Finally, hydrochloric acid and alcohol were added for decolorization. After decolorization, the insects were dehydrated with 85%, 95%, and 100% ethanol, sealed with gum, dried, and placed under a Nikon orthotopic microscope and confocal laser microscope to observe gonadal changes.

### Histological evaluation

Fresh liver tissue was fixed overnight in 4% paraformaldehyde. After dehydration, the tissues were paraffin-embedded into 4 mm sections and subjected to hematoxylin, eosin (HE), and Masson staining (Hu et al., 2020). The formerly observed changes in the formation of egg granulomas and the last observed changes in the deposition of collagen fibers were documented, in which collagen fibers were stained blue and the background was stained red. The areas featuring granulomas and fibrosis surrounding single eggs and glance were observed at 200× and 20× magnification.

### Statistical analysis

The data analysis performed was in the form of means ± standard deviation (SD). All results were analyzed and statistically plotted using GraphPad Prism 9.0 software. An independent sample Student’s *t-test* was used to assess differences between the two groups, and a *p* < 0.05 was considered to indicate statistical significance.

## Results

### *Schistosoma japonicum* encodes two metabotropic glutamate receptors, one of which is non-canonical

**FIGURE 1:**
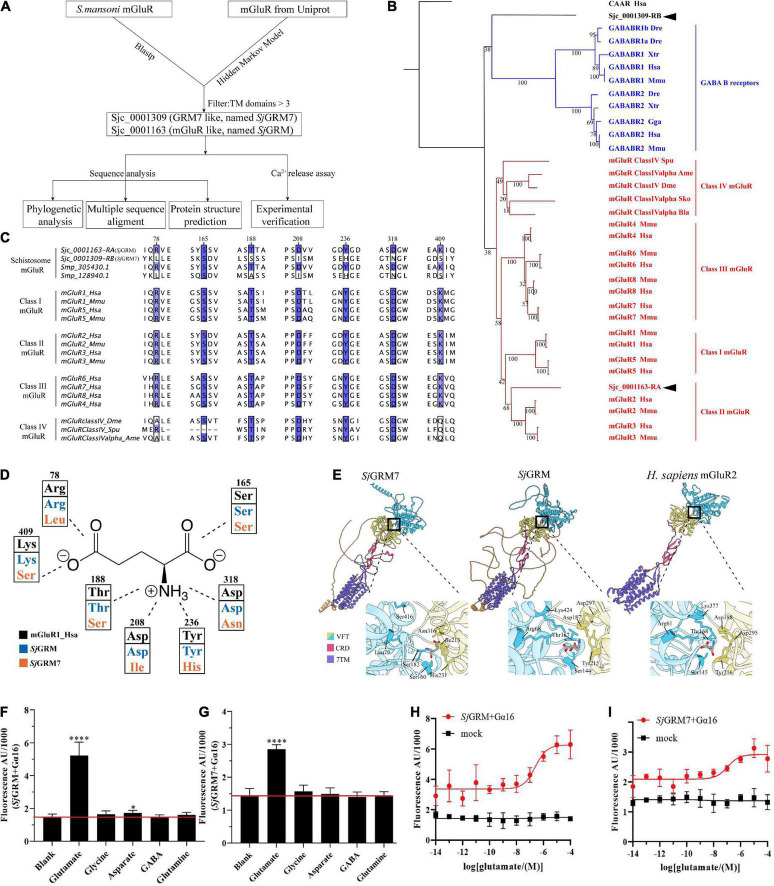
Bioinformatic analysis reveals *Sj*GRM7 as a schistosome-specific mGluR. **(A)** A schematic for bioinformatics and functional analysis of *S. japonicum* mGluR. **(B)** mGluR maximum-likelihood tree. mGluRs and GABAB receptors (GABABR) are indicated by the branches and text colors. The text on the right shows further classification. Two *S. japonicum* mGluRs are highlighted in black. The species used in this analysis are implicated using a three-letter species code, except for *S. japonicum* (Sjc), *S. mansoni* (Smp), and *Schmidtea mediterranea* (SMESG). Phylogenetic reconstruction was performed using the maximum-likelihood method with the WAG + I + G + F model and an ultrafast bootstrap of 1,000 iterations. Bootstrap values are shown at the tree nodes, and protein names are shown at the end of each branch. The human calcium-sensing receptor was used as an outgroup. The scale bar denotes the number of amino acid substitutions per site. **(C)** Multiple protein alignments of mGluR ligand-binding residues. Representative sequences were chosen for each class with labels on the left. Key residues are colored according to percentage identity. The residue numbers indicated at the top correspond to those in human mGluR1. **(D)** Schematic depiction of mGluR’s key residues involved in ligand binding. Human mGluR key residues and those of *S. japonicum* are shown for comparison. As shown in panel **(C)**, the residue and its number corresponded to multiple protein alignments. Residues are colored based on proteins, as shown in the legend. The dashed line indicates the part of the glutamate the residue that interacts with other residues. **(E)** Protein structure comparisons between the predicted two mGluRs of *S. japonicum* structures and human mGluRII crystal structures. Different classes corresponded to other protein domains, as shown in the legend. Orange structures are intra-cellular regions, but most have low confidence (pLDDT < 50). Dashed lines in human mGluR2 denote disordered fragments. The binding pockets are shown by black boxes and are zoomed at the bottom right of each structure. Key residues involved in ligand binding are depicted in close-up views using the residue number and three-letter code. **(F)** *Sj*GRM-expressing HEK293 cells were treated with various amino acid transmitters (L-glutamate, glutamine, GABA, glycine, and aspartate) at 10^– 4^ M or vehicle (blank). **(G)** *Sj*GRM7-expressing HEK293 cells were treated with various amino acid transmitters (L-glutamate, glutamine, GABA, glycine, and aspartate) at 10^– 4^ M or vehicle (blank). **(H)** *Sj*GRM-expressing HEK293 cells were treated with different concentrations of L-glutamate, and the vector (blank) was plotted in a dose-dependent manner. **(I)** *Sj*GRM7-expressing HEK293 cells were treated with different concentrations of L-glutamate, and the vector (blank) was plotted in a dose-dependent manner. mGluR and GRM, metabotropic glutamate receptor; HEK293, human embryonic kidney 293T cells, GABA, γ-aminobutyric acid. *P < 0.05, ****P < 0.0001.

In this section, we validated the existence of two mGluRs in *S. japonicum* (Figure 1, top workflow). We identified two mGluRs, Sjc_0001309 (similar to GRM7, named *Sj*GRM7) and Sjc_0001163 (similar to mGluR, named *Sj*GRM), by combining Blastp and HMM models, and those with fewer than three TM structural domains were filtered out. A phylogenetic study was performed next. The Bayesian and ML trees were in agreement that *Sj*GRM belongs to class II GRM and that *Sj*GRM7 is in a distinct position close to the GABA B family (Figure 1B). Except for SMESG000022915001, the homolog of *Sj*GRM7 in *Schmidtea mediterranea*, which is a Class IV GRM, the homologs in *S. mansoni* and *Schmidtea mediterranea* are in the same phylogenetic position.

The glutamate binding of GRM requires the binding of α-carboxy, α-amino, and γ-carboxyl groups. This is achieved using seven conserved amino acid residues, as shown in Figure 1C. The function of mGluR is severely damaged if any one of the seven residues mutates. According to the multiple sequence alignment (Figure 1C), *Sj*GRM contains all the conserved amino acids involved in ligand binding. However, *Sj*GRM7 contains only a conserved serine (160 in *Sj*GRM7), which is responsible for binding the α-carboxy group of glutamate (Figures 1C,D). None of the other conserved amino acids was present in *Sj*GRM7. This is the same for its homolog in *S. mansoni* Smp_128940, where six out of the seven essential amino acids differ from the conserved residues (Figure 1C). Additionally, Smp_128940 contains an alanine (183 in Smp_128940) rather than a serine in *Sj*GRM7 (183 in *Sj*GRM7). Despite the differences in conserved residues, Smp_128940 can still be activated by L-glutamate (Taman and Ribeiro, 2011). Protein structures were further predicted by examining the spatial arrangement of critical residues. Both *Sj*GRM and *Sj*GRM7 have typical GRM domains, including a VFT for ligand binding, a cysteine-rich linker domain, and a seven-transmembrane domain (Figure 1E). The glutamate docking results showed similar conserved residue positions between *Sj*GRM and human GRM2. However, Leu79 and Ser416 in *Sj*GRM7 are absent from the binding pocket. Furthermore, no other visible residue close to the pocket could replace leucine and serine to bind the γ-carboxyl group.

To confirm that the two mGluRs obtained using a bioinformatics approach can specifically bind glutamate, their ability to bind glutamate was determined using a live intracellular calcium mobilization assay. The cDNAs of these two mGluRs were transiently transfected into HEK293 cells with the heterotrimeric G-protein Gα16 to detect intracellular calcium mobilization. The results showed that *Sj*GRM and *Sj*GRM7 responded only to L-glutamate but not to glutamine (glutamate derivative), γ-aminobutyric acid (GABA), glycine, or aspartate (Figures 1F,G), and both showed a dose-dependent relationship with L-glutamate (Figures 1H,I). This result further verifies that *Sj*GRM and *Sj*GRM7 are glutamate receptors.

Our results showed that *S. japonicum* encodes two mGluRs, Sjc_0001163 and Sjc_0001309, referred to as *Sj*GRM7 and *Sj*GRM, respectively. *Sj*GRM has all the typical glutamate receptor features, and *Sj*GRM7 can recognize glutamate despite differences in phylogeny, key residues, and binding pockets.

### Localization and expression of the metabotropic glutamate receptors in *Schistosoma japonicum*

To further describe these two mGluRs and enable future research on their physiological functions, two of mGluRs’ mRNA expression changes during different *S. japonicum* developmental stages were quantified, and their cellular location was observed.

**FIGURE 2:**
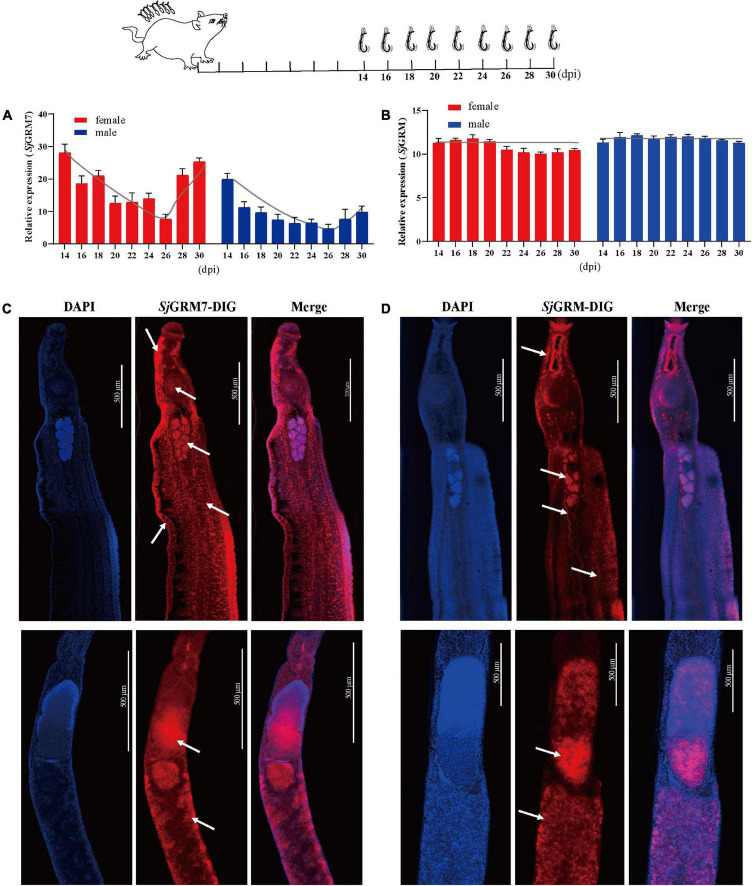
Localization and expression of the two mGluRs in *S. japonicum.* The top is a flow chart of the samples collected during the different developmental periods and most worms in 14 and 16 dpi are not paired. **(A)** Expression patterns of *Sj*GRM7 at different time points post-infection. **(B)** Expression patterns of *Sj*GRM at different time points post-infection. **(C)** Sagittal confocal section of FISH showing *Sj*GRM7 mRNA enriched in the testes, brain ganglia, longitudinal neuraxis, ventral suckers, and peripheral nerve cells of a male worm (top) and in the ovaries and vitelline glands of a female worm (bottom) (white arrows). **(D)** Sagittal confocal section of FISH showing *Sj*GRM mRNA enrichment in the testes of male worms (top) and in the ovaries and vitelline glands of female worms (bottom). FISH analysis of the locations of the two *S. japonicum* mGluRs. Nuclei were stained blue (DAPI), and dig-labeled mGluRs were stained red. Fifteen parasites were used in the five experiments. Specific signals are indicated by white arrows. Scale bar: 500 μm **(C,D)**. FISH, fluorescence *in situ* hybridization; mGluR and GRM, metabotropic glutamate receptor; DAPI, 4′,6-diamidino-2-phenylindole.

The expression of *Sj*GRM and *Sj*GRM7 at different developmental stages (14, 16, 18, 20, 22, 24, 26, 28, and 30 dpi) was examined by qPCR. Among them, most worms in 14 and 16 dpi are not paired. The results showed that the expression of *Sj*GRM was relatively stable during *S. japonicum* development (Figure 2B). *Sj*GRM7 was first downregulated before 26 dpi and then upregulated after 26 dpi at different developmental stages of *S. japonicum* (Figure 2A).

The *Sj*GRM has specific signals in the testes of male and female ovaries, notably in mature oocytes (Figure 2D, white arrows), whereas its fluorescence intensity is not as strong as *Sj*GRM7. Meanwhile, there are no specific signals in the *Sj*GRM for longitudinal neuraxis and peripheral nerve cells. As a result, *Sj*GRM7 may be crucial for the growth of *S. japonicum*.

### *Sj*GRM7 is required for normal physiological activity in *Schistosoma japonicum in vitro*

To observe the function of the two mGluRs in *S. japonicum* and their phenotypes in real time, we performed RNA interference (RNAi) in adult paired worms *in vitro*.

**FIGURE 3:**
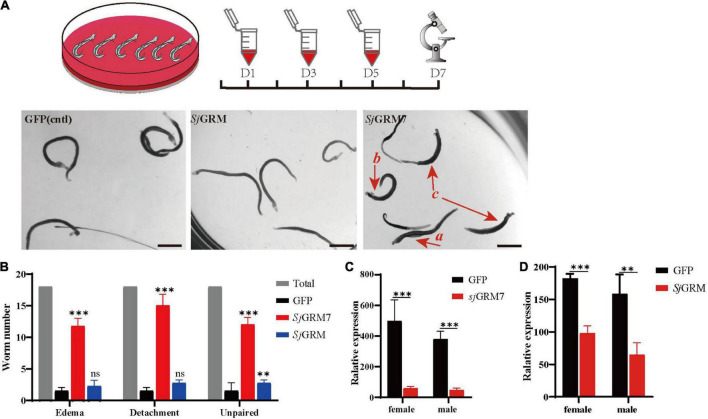
*Sj*GRM7 is required for normal physiological activity in *S. japonicum in vitro*. The top is the flow chart of *in vitro* interference. Adults with good pairing activity were added to the petri dish, dsRNA was added for interference on the first, third, and fifth days, and microscopic observation and photography were carried out on the seventh day. Each plate had six pairs of three replicate wells for each biological experiment with four biological replicates. **(A)** Observation under the light microscope on the seventh day after dsRNA treatment of the control (left), *Sj*GRM (middle), and *Sj*GRM7 (right) groups. Scale bar: 200 μm. **(B)** Effect statistics of the control, *Sj*GRM, and *Sj*GRM7 groups after dsRNA treatment, with the statistical indicators of swelling (detachment), no adsorption to the chassis, and unpairing. qPCR was used to detect the mRNA expression of *Sj*GRM7 **(C)** and *Sj*GRM **(D)** after treatment with dsRNA. ^***^P < 0.001, ^**^P < 0.01. mGluR and GRM, metabotropic glutamate receptor; qPCR, reverse transcription polymerase chain reaction; dsRNA, double-stranded RNA.

First, the dsRNAs were generated and then added on days 1, 3, and 5 using the immersion method, and microscopic observation was performed on day 7 (see Figure 3, top). The results showed no significant change in phenotype in the *Sj*GRM dsRNA group compared with the GFP control group. Conversely, the *Sj*GRM7 dsRNA group failed to attach to the petri dish, and the paired worms showed separation and edema (Figure 3A, red arrows). The number of worms that appeared for each phenotype was observed, recorded, and subjected to statistical analysis (Figure 3B).

The *Sj*GRM7 dsRNA group showed the highest degree of edema, detachment of the chassis, and separation of paired worms (65.28%, 83.33%, 66.67%, respectively), followed by the *Sj*GRM dsRNA group (12.5%, 15.28%, 15.28%, respectively) and the GFP control group (8.33%, 5.56%, 11.11%, respectively) (Figure 3B). RNAi efficiency was measured using qPCR to demonstrate that RNAi caused this phenotype in *Sj*GRM7 and *Sj*GRM. The results confirmed that both male and female *Sj*GRM7 and *Sj*GRM genes were knocked down (Figure 3C). These results suggested that *Sj*GRM7 is critical for the maintenance of regular physiological activity in *S. japonicum*.

### *Sj*GRM7 affects development and reproduction in *Schistosoma japonicum* and reduces worm burden *in vivo*

**FIGURE 4:**
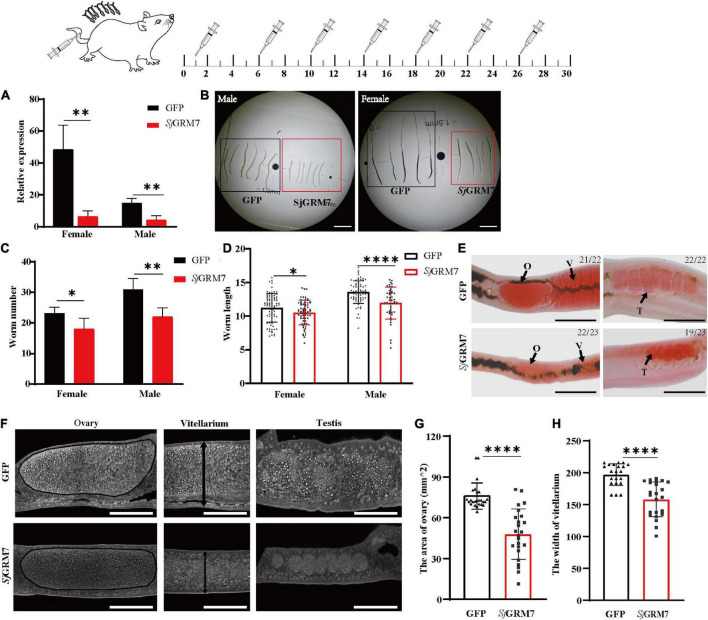
Effects of dsRNA-mediated knockdown of *Sj*GRM7 *in vivo.* The top is a flow chart of *in vivo* interference. On the 1st, 6th, 10th, 14th, 18th, 22nd, and 26th days after cercariae infection using the abdominal patch method, dsRNA was injected through the tail vein, and the hepatic portal vein was perfused on the 30th day for observation. Worms were continuously treated with 10 μg *Sj*GRM7 and GFP dsRNA *in vivo*. **(A)** *Sj*GRM7 transcript relative mRNA levels in the *Sj*GRM7 dsRNA group. **(B)** Morphological changes in the worms captured at 30 dpi. **(C)** Comparison of worm numbers between GFP and *Sj*GRM7 dsRNA groups. **(D)** Comparison of worm length between the GFP and *Sj*GRM7 dsRNA groups. **(E)** Morphological changes in the gonads of female and male worms were observed by carmine staining. **(F)** Adult worms from the treatment group were analyzed by confocal laser scanning microscopy. **(G)** The area of the largest cross-section of the ovary of female worms was measured using ImageJ. **(H)** The width of the largest cross-section of the vitellarium of female worms was measured using the ImageJ software. O, oocytes; E, eggs; T, testis; GFP, green fluorescent protein; dpi, days post infection; dsRNA, double-stranded RNA; GRM, metabotropic glutamate receptor. Scale-bars: **(E)** 200 μm; **(F)** 50 μm. ^*⁣*⁣**^P < 0.0001, ^**^P < 0.01, *P < 0.05.

Since we showed that *Sj*GRM7 affects the normal physiology of *S. japonicum, Sj*GRM7 was further investigated. We examined the *Sj*GRM7 function to investigate whether it also affects the normal physiology of *S. japonicum* in the definitive host. The level of *Sj*GRM7 mRNA was first detected using qPCR after interference. The results showed that the mRNA levels were significantly downregulated in the *Sj*GRM7 dsRNA group compared to the GFP control group, with 86.92% and 72.04% downregulation in females and males, respectively (Figure 4A).

To study the morphological effects of *Sj*GRM7 on *S. japonicum*, brightfield microscopy was used to image and quantify worm morphology in mice. Compared to the GFP control group, the growth of male (Figure 4B, left) and female (Figure 4B, right) worms in the *Sj*GRM7 dsRNA group was significantly inhibited (Figure 4B). ImageJ was used to measure the length of female and male worms, and the length shortening degrees of females and males were 12.09% and 6.1%, respectively (Figure 4D). Quantification results showed that the *Sj*GRM7 dsRNA group had significantly reduced worm loads. The degrees of reduction in female and male worms were 22.58% and 29.03%, respectively (Figure 4C). These data indicate that the interference of *Sj*GRM7 can affect the development of *S. japonicum* and may even have an insecticidal effect. However, *Sj*GRM knockdown has no influence on *S. japonicum* growth and development (Supplementary Figure 1).

To further characterize the gonadal development of *S. japonicum* after *Sj*GRM7 interference, *S. japonicum* was stained with carmine, followed by visualization of changes in the gonads using fluorescence microscopy, and changes at the cellular level using laser confocal microscopy. Morphological observations showed that the GFP control group more mature ovaries, vitelline glands, and testes, which were significantly larger than those of the *Sj*GRM7 dsRNA group (Figure 4E). To further observe the development of the reproductive system at the cellular level, we used confocal laser microscopy to observe the gonads of *S. japonicum*. The results showed that the GFP control ovaries were larger and filled with more mature oocytes. In comparison, ovaries of the *Sj*GRM7 dsRNA group were smaller and contained a smaller number of immature oocytes. We also observed that the GFP control group had far more mature vitelline gonad cells than the *Sj*GRM7 dsRNA group (Figure 4F, left, middle). The male testicular area and testicular spermatocytes were significantly reduced in the *Sj*GRM7 dsRNA group (Figure 4F, right).

We also measured the ovary area and maximum cross-sectional width of the vitelline gland to quantify the level of female gonad development. The results showed that sexual maturation was significantly inhibited in the ovaries (Figure 4G) and vitelline glands (Figure 4H) of females in the *Sj*GRM7 dsRNA group, with 35.62% and 19.65% inhibition, respectively. These results suggested that *Sj*GRM7 is essential for the growth, development, and survival of *S. japonicum*.

### *Sj*GRM7 affects oviposition in *Schistosoma japonicum* and alleviates host pathology *in vivo*

**FIGURE 5:**
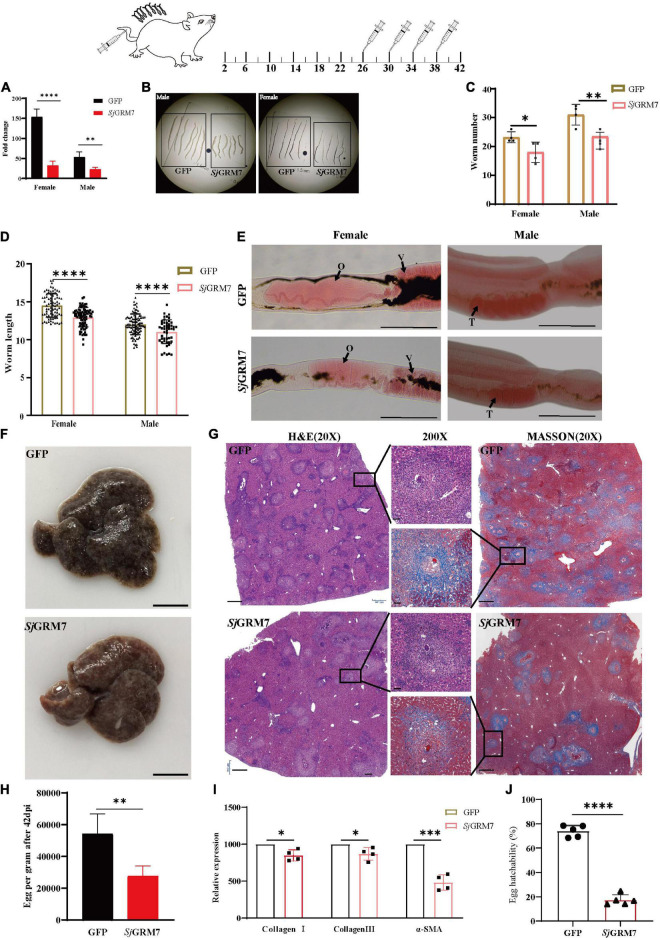
*Sj*GRM7 affects oviposition in *S. japonicum* and alleviates host pathology *in vivo.* On the 26th, 30th, 34th, and 38th days after abdominal patch infection, dsRNA was injected through the tail vein, and the hepatic portal vein was perfused on the 42nd day. **(A)** *Sj*GRM7 transcript relative mRNA levels in the *Sj*GRM7 dsRNA group. **(B)** Morphological changes in worms collected at 42 dpi. **(C)** Comparison of worm numbers between GFP and *Sj*GRM7 dsRNA groups. **(D)** Comparison of worm length between the GFP and *Sj*GRM7 dsRNA groups. **(E)** Morphological changes in the gonads of female and male worms were observed by carmine staining. **(F)** Gross observations of the mouse liver in the GFP and *Sj*GRM7 dsRNA groups. **(G)** Liver sections stained with hematoxylin and eosin (H&E) (left) showing egg granulomatous lesions; Masson’s trichrome staining (right); liver fibrosis, granuloma formation, and liver fibrosis from individual eggs were also observed (middle). **(H)** Eggs per gram comparison between the GFP control and *Sj*GRM7 dsRNA groups. **(I)** qPCR showed that the mRNA levels of collagen I, collagen III, and α-SMA in the *Sj*GRM7 dsRNA group were significantly lower than those in the GFP control group. **(J)** Comparison of egg hatchability between GFP and *Sj*GRM7 dsRNA groups. O, oocytes; e, eggs; T, testis. E, 200 μm; F, 10 mm.; G, left and right, 500 μm; middle, 50 μm; GFP, green fluorescent protein; dpi, days post infection; dsRNA, double-stranded RNA; GRM, metabotropic glutamate receptor; qPCR, quantitative polymerase chain reaction; α-SMA, α-smooth muscle actin protein ^*⁣*⁣**^P < 0.0001, ***P < 0.001, ^**^P < 0.01, *P < 0.05.

The results in Figure 4 indicate that *Sj*GRM7 is necessary for maintaining the normal physiology of *S. japonicum* and plays an essential role in its growth and development. We further investigated the effect of *Sj*GRM7 on egg-laying by *S. japonicum* after sexual maturation (Figure 5, top).

Downregulation of *Sj*GRM7 mRNA levels after interference was first detected by qPCR, with 78.99% and 57.32% for females and males, respectively (Figure 5A). Growth of female and male *S. japonicum* was inhibited in the *Sj*GRM7 dsRNA group (Figure 5B), with inhibition lengths of 10.71% and 10.92%, respectively (Figure 5D) (ImageJ quantification). The loads were reduced by 17.20% and 20.59% in females and males, respectively (Figure 5C).

Observations of the gonads after carmine staining showed that female ovaries of the *Sj*GRM7 dsRNA group were significantly smaller than those of the GFP control group. The morphology of the male testes was significantly smaller than that of the GFP control group (Figure 5E). These results indicate that RNAi affects the development of *Sj*GRM7 and has a repellent effect on *S. japonicum* after maturation up to egg-laying.

We further investigated whether this affected gonadal function in female worms as well as egg-laying and immunopathology of liver damage caused by worm egg antigens. Many studies have shown that liver fibrosis results from an interaction between liver parenchymal cell damage and hepatic stellate activation. The proliferation and activation of stellate cells plays a major role in liver fibrosis. Activated hepatic stellate cells express α-smooth muscle actin protein (α-SMA), and extracellular matrix proteins, mainly collagen I and collagen III, accumulate to form fibrotic scarring that gradually leads to liver fibrosis.

Therefore, we first directly observed the livers after dissecting the mice and found that the livers of the *Sj*GRM7 dsRNA group had only a few nodules formed by worm eggs. In contrast, the livers of the GFP control group exhibited a severe degree of fibrosis (Figure 5F). We quantified the number of eggs per gram of liver tissue. We showed that the *Sj*GRM7 dsRNA group had significantly fewer eggs than the GFP control group, with a reduction of 48.98% (Figure 5H), while at the same time we also observed the hatching rate of eggs. The results showed that the hatching rate of the *Sj*GRM7 dsRNA group was 76.63% lower than that of the control group (Figure 5J and Supplementary Figure 2). We hypothesized that the reduction in liver damage in the *Sj*GRM7 dsRNA group may have been caused by reduced egg production and a decrease in egg activity.

To further observe the liver pathology of mice, HE and Masson staining were used to observe the reaction process of liver egg granulomas and liver fibrosis, respectively (Figure 5G). HE staining results (Figure 5G, left) showed that the number of granulomas formed by eggs in the *Sj*GRM7 dsRNA group was lower than that in the control group, and the area of granulomas formed by individual eggs was also significantly smaller (Figure 5G, middle). Masson staining (collagen fibers in blue), which characterizes the pathology of hepatic fibrosis (Figure 5G, right), showed that the *Sj*GRM7 dsRNA group had significantly less hepatic fibrosis than the GFP control group, and the area of hepatic fibrosis formed by single eggs was also significantly lower. To quantify the extent of fibrosis, we used qPCR to measure changes in the mRNA levels of α-SMA, collagen I, and collagen III during liver fibrosis. The results showed that the mRNA levels of the three genes were significantly reduced in the *Sj*GRM7 dsRNA group compared to those in the GFP control group, with reductions of 14.92%, 12.98%, and 51.67%, respectively (Figure 5I). The pathological section and qPCR results suggest that *Sj*GRM7 interference led to a reduction in liver damage. These results suggested that *Sj*GRM7 interference could reduce pathologically damage to the host by affecting the development, egg production, and egg activity of *S. japonicum*.

### RNA-seq analysis shows that knocking down *Sj*GRM7 leads to downregulation of the G protein-coupled receptor signaling pathway in *Schistosoma japonicum*

The results in Figure 5 show that *Sj*GRM7 plays an essential role in maintaining the normal physiology, growth, development, and egg production of schistosomes. To further explore the regulatory mechanism of *Sj*GRM7 expression in the development of *S. japonicum*, we performed RNA-seq analysis of both male and female worms after knocking down *Sj*GRM7 *in vitro*.

**FIGURE 6:**
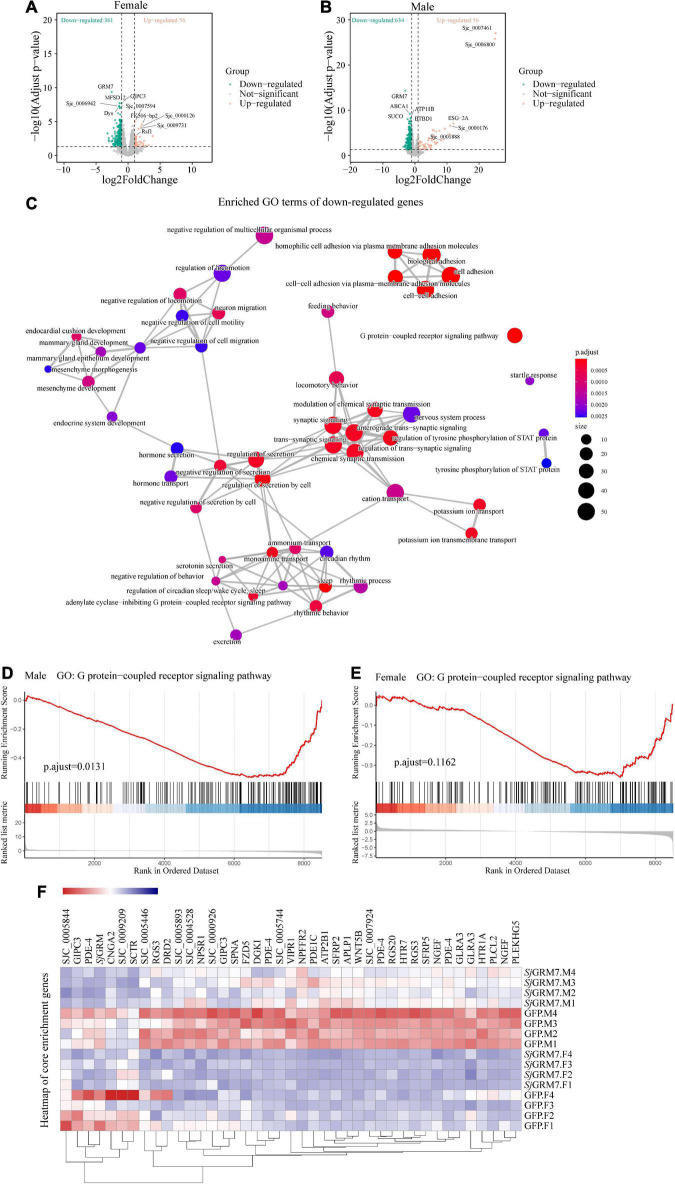
Differentially expressed genes (DEGs) of *S. japonicum* after *Sj*GRM7 RNAi *in vitro* and gene ontology (GO) analysis. **(A)** DEG volcano plots in females and males. **(B)** *X*-axis: log_2_-fold change (ds *Sj*GRM7/GFP). *Y*-axis: log_10_ (adjusted *p*-value). Green points represent significantly downregulated genes, and orange points represent significantly upregulated genes. **(C)** GO term visualization (enrichment calculated using gene-set enrichment analysis, *p* < 0.05, adjusted for FDR, only shown in genes significantly downregulated after *Sj*GRM7 knockdown). **(D)** GSEA showed downregulation of the GPCR signaling pathway in *Sj*GRM7 dsRNA group male worms and female worms **(E)** as compared to GFP controls. **(E)** Heat map of core enrichment genes in both males and females for the GPCR signaling pathway gene set. **(F)** The score at the peak of the plot **(D,E)** is the enrichment score (ES) for this gene set, and genes that appear before or at the peak are defined as core enrichment genes for this gene set. GFP, green fluorescent protein; dsRNA, double-stranded RNA; GRM, metabotropic glutamate receptor; GPCR, G protein-coupled receptor; GSEA, Gene set enrichment analysis; FDR, false discovery rate.

We set log_2_FoldChange > 1 (upregulated), log_2_FoldChange < **−**1 (downregulated), and false discovery rate (FDR) < 0.05, as cutoffs for differential gene expression analysis. We noticed that knockdown of *Sj*GRM7 had strong effects on gene expression, especially the downregulation of gene expression. The overall expression profile of RNAi versus the control revealed 361 downregulated and 56 upregulated genes in females and 634 downregulated and 56 upregulated genes in males (Figures 6A,B). After the knockdown of *Sj*GRM7, genes with the most significant expression changes included *ABCA1* (Sjc_0002054), *ATP11a* (Sjc_0009436), *BTBD1* (Sjc_0002226), *SUCO* (Sjc_0002288), and *ELAVL1* (Sjc_0002420) in males, and *GIPC3* (Sjc_0008441), *MFSD12* (Sjc_0005954), *Dys* (Sjc_0003555), and *Sjc_0006942* in females. Supplementary Figure 3 also shows that validation of genes predicted by RNA-seq to change substantially using qPCR. Thus, *Sj*GRM7 may regulate these genes. GO analysis of the downregulated genes in male worms revealed that *Sj*GRM7 knockdown had powerful effects on cell-cell adhesion (GO:0098609), GPCR signaling pathway (GO:0007186), secretion regulation by cells (GO:1903530), and modulation of chemical synaptic transmission (GO:0050804) (Figure 6C and Supplementary Table 1). However, we only observed significant enrichment of genes related to axonemal dynein complex assembly (GO:0070286) in females (Supplementary Table 2). Considering that the expression of certain genes may only change slightly, and these are not identified as differentially expressed genes after RNAi, we performed gene set enrichment analysis (GSEA) (Supplementary Tables 3, 4). By comparing the results obtained in females and males separately, we observed that several genes involved in synaptic signaling (GO:0099536), chemical synaptic transmission (GO:0007268), GPCR signaling pathway (GO:0007186), cyclic-nucleotide-mediated signaling (GO:0019935), axon development (GO:0007409), muscle organ development (GO:0007517), endocrine system development (GO:0035270), and regulation of locomotion (GO:0040012) were downregulated in females and males (Supplementary Table 5). This result is highly consistent with functional studies of *Sj*GRM7 in other species (Su et al., 1998; Song et al., 2021). This suggests that *Sj*GRM7 plays an essential role in the neuronal development of *S. japonicum.*

Among these pathways, we noted significant downregulation of the neural-associated endocytic adaptor protein Numb (Sjc_0007478), the first identified cell fate determinant in *Drosophila melanogaster* (Shan et al., 2018). Studies have shown that Numb proteins are present in post-mitotic neurons. *In vitro* studies have shown that Numb is involved in neuronal morphological development, such as synaptic growth and spine development, and that deletion of Numb/Numblike in glutamatergic neurons leads to anxiety-like behavior in mice (Qian et al., 2017). Deficiency of Numb protein impairs prominent mGlu1 expression and motor coordination. This leads to impaired transport of mGlu5 and autistic-like behavior in the neurons (Wang et al., 2019). Additionally, Numb enhances Notch by inhibiting the ubiquitination of the intracellular structural domain of neurogenic locus notch homolog protein 1 (NOTCH1) signaling (Luo et al., 2019).

Furthermore, to understand the effect of *Sj*GRM7 knockdown on the expression of other GPCRs in *S. japonicum*, we examined the gene expression levels in GPCR gene sets that were significantly enriched in both sexes (Figures 6D,E). Other GPCR genes that significantly downregulated in male worms included sex peptide receptor (Sjc_0000420), tachykinin-like peptide receptor 99D (Sjc_0003380), Dro/myosuppressin receptor (Sjc_0007429), neuropeptide FF receptor 2 (Sjc_0003026), FMRFamide receptor (Sjc_0006030), PDF receptor (Sjc_0000792), and other neuropeptide GPCR proteins. Interestingly, the gene expression of neuroendocrine protein 7b2 (Sjc_0001267), a canonical molecular marker of developing and definitive neurons, was notably downregulated after the knockdown of *Sj*GRM7. This suggests that knockdown of *Sj*GRM7 may affect the development of neurons, leading to a deregulated phenotype in central nervous system function. In female worms, the most significantly downregulated GPCRs were the 5-hydroxytryptamine receptor (Sjc_0005944), dopamine D2-like receptor (Sjc_0009336), octopamine receptor (Sjc_0000926), 5-hydroxytryptamine receptor 7 (Sjc_0001525), secretin receptor (Sjc_0000311) and other biogenic amine receptors.

As shown in Figure 6F, most GPCRs were expressed at higher levels in males than in females. We speculate that the sex differences in their expression levels led to our observation that these genes were significantly downregulated in males after *Sj*GRM7 knockdown but only slightly downregulated in females.

## Discussion

Functional genomics has paved the way for drug discovery to combat schistosomiasis. Extensive efforts have been made to characterize the genes essential for development and maintenance (Wang et al., 2020), especially in the neural and reproductive systems. These are potential targets of the only effective drug, praziquantel (Love et al., 2014; Park et al., 2021), and the root of schistosome pathology. Glutamate is an essential neurotransmitter in worms that interacts with various cell surface receptors to transmit excitatory signals. GRMs are one of the most important classes of glutamate receptors.

Interestingly, *Sj*GRM7 changed six out of seven conserved binding residues but retained its function and selectivity. This receptor can only be activated by L-glutamate but not by GABA, glycine, aspartate, or the glutamate derivative glutamine (Figure 1E). This is unexpected because previous work with mutagenesis showed that, except for K409 (in human GRM1), mutations in any of these key residues severely hinder the receptor’s ability to bind glutamate (Hara et al., 1993; Sato, 2003). Glutamate and the orthosteric agonist quisqualic acid potency are reduced 1,000- and 100-fold, respectively, in an R78L rat GRM1 mutant (Jensen et al., 2000). Nevertheless, a R78L mutation was present in *Sj*GRM7 and Smp_128940. This inconsistency challenges the current understanding of the mGluR-binding pocket. Only one conserved residue (serine183 in *Sj*GRM7) is unlikely to be involved in glutamate binding. Therefore, unreported residues in the binding pocket may be involved in binding. Three-dimensional (3D) structure determination of the binding pocket and mutagenesis of the associated residues are required to further explain the unique binding mechanism of *Sj*GRM7. In addition, this unique binding pocket is an optimal target for drug discovery, as this difference in *Sj*GRM7 will allow more accessible screening of selective orthosteric chemistries due to the highly conserved glutamate-binding mechanism while minimizing potential side effects.

We think *Sj*GRM7 may have acquired new functions other than typical mGluR’s functions, and this new function may play a more important role in the growth and development of schistosomes than *Sj*GRM. Having most of the conserved residues mutated and being evolutionarily distinct from the mGluR group may be signs of it gaining new functions. The previous report shows that the canonical mGluR knockdown only causes a neurodevelopmental disorder phenotype without impairing its growth, development and survival (Fisher et al., 2018). Thus, it can be implied that the knockdown of mGluRs in schistosomes will produce similar phenotypes. In the present study, the *Sj*GRM knockdown worms only show unpairing *in vitro.* No effect on growth, development and survival was detected *in vivo*, consistent with the results of typical mGluR after knockdown. In contrast, the uncanonical *Sj*GRM7 knockdown worms showed edema, non-absorption of chassis, and separation of paired worms *in vitro.* It also shows an effect on maturation, development, survival, egg production, and activity of *S. japonicum in vivo*, which in turn significantly attenuated the pathological damage to the host. We reason this discrepancy may be caused by *Sj*GRM7 acquiring new functions in the time of evolution beyond merely modulating neurotransmission. It also further suggests that uncanonical *Sj*GRM7 is important and more likely to be a target for a novel anti-schistosomal drug.

Another striking result from our RNA-seq data is the significant downregulation of glioma-Associated oncogene homolog 1 (*GLI1*) and non-ribosomal peptide synthetase (*NRPS*) expression in both females and males, which is essential for the male-derived non-ribosomal peptide pheromone control of female schistosome development (Chen et al., 2022). Combined with this phenotype, we observed *Sj*GRM7 knockdown *in vivo* and *in vitro*. *In vivo*, *Sj*GRM7 knockdown caused abnormal growth in both male and female mice. However, we only observed an abnormal male phenotype after knocking down *Sj*GRM7 *in vitro*. These results suggest that *Sj*GRM7 may play a critical regulatory role, mainly in nervous system development and synaptic transmission in males.

*In situ* hybridization results showed that *Sj*GRM7 was expressed in neurons and testes in males and only in gonads in females (Figures 2C,D), which matched the abnormal male phenotype observed only after *in vitro* knockdown of *Sj*GRM7. Studies have shown that in *Macaca fascicularis*, GRM2 is located in primordial follicles and oocytes (Gill et al., 2008). BLAST was used to identify genes with approximately 83% similarity to *Sj*GRM7 and *Sj*GRM in *S. mansoni*, Smp_128940, and Smp_305430, respectively. The locations of these two genes were predicted using the *S. mansoni* single-cell sequencing website (Wendt et al., 2021). The results showed that both were expressed throughout the body, with Smp_305430 mainly expressed in neurons in both sexes and in the muscle of females. Smp_128940 was mainly expressed in neurons and germ cells (Supplementary Figure 4). Smp_128940, is expressed in Germline Stem Cell (GSC) zygotes and late germinating cells, suggesting a conserved role in GSC regulation (Taman and Ribeiro, 2011; Wendt et al., 2020). We speculate that the combined effect of *Sj*GRM7 knockout in females and decreased levels of transmitted non-ribosomal peptide pheromones are due to the downregulation of *GLI1* and *NRPS* genes in males which causes abnormal development in females. In humans, mGluRs mainly affects the central nervous system and are associated with neurodegenerative diseases (Fabiola et al., 2017; Ribeiro et al., 2017). Nevertheless, GPCR signaling plays an important role in germ cell regulation. In *Drosophila melanogaster* neuropeptide Y receptor 1 (NPYR1) in neuroendocrine cells receives signals from NPY8, which in turn regulates germ cell maturation (Amir et al., 2016).

In conclusion, we identified two *S. japonicum* mGluRs: *Sj*GRM and *Sj*GRM7. *Sj*GRM7 diverged from typical mGluRs at an early time point and may have a novel binding pocket. This receptor is expressed in neural and reproductive systems. Knockdown of *Sj*GRM7 affects worm development, viability, and reproduction while dramatically reducing pathology. Further, RNA-seq results demonstrated downregulation of the GPCR pathway, neural function, and cell adhesion. This study presents a promising drug target for schistosomiasis, and further research on *Sj*GRM7 may improve our understanding of sexual gonad maturation and maintenance.

## Data availability statement

The sequencing data were deposited in the NCBI Sequence Read Archive (SRA) database under accession numbers (PRJNA880502).

## Ethics statement

Animal studies were reviewed and approved by the Laboratory Animal Welfare Review Committee of the Institute for the Prevention and Control of Parasitic Diseases (National Center for Tropical Diseases Research) of the Chinese Center for Disease Control and Prevention (approval number: IPD-2020-10), and did not involve informed consent was not obtained.

## Author contributions

XW, WZ, YX, WL, YY, CY, BZ, and MG conducted experiments. XW, BX, JW, YL, and WH designed the experiments and analyzed the data. SC and XC analyzed the bioinformatic data. WZ, XC, MG, and WL provided intellectual input and aided in experimental design. XW wrote the manuscript. WH revised the manuscript accordingly. All authors have contributed to the manuscript and approved the submitted version.

## Footnotes

1
https://cran.r-project.org/web/packages/pheatmap/index.html

2
https://imagej.nih.gov/ij/

## Supplementary material

Supplementary Table 1 ds*Sj*GRM7_M_VS_dsGFP_M_GOterms_enrichment.

Supplementary Table 2 ds*Sj*GRM7_VS_dsGFP_F_GOterms_enrichment.

Supplementary Table 3 ds*Sj*GRM7_VS_dsGFP_M_GSEA_enrichment.

Supplementary Table 4 ds*Sj*GRM7_VS_dsGFP_F_GSEA_enrichment.

Supplementary Table 5 GSEA_shareGOBPs.

Supplementary Table 6 All primers.
