# Supplementary material for: A metabotropic glutamate receptor affects the growth and development of Schistosoma japonicum
Source: Front Microbiol. 2022 Nov 30;13:1045490. doi: 10.3389/fmicb.2022.1045490 (PMC9750798; doi:10.3389/fmicb.2022.1045490)
Supplement: Supplementary file 1 [file Presentation_1.PPTX]

## Slide 1
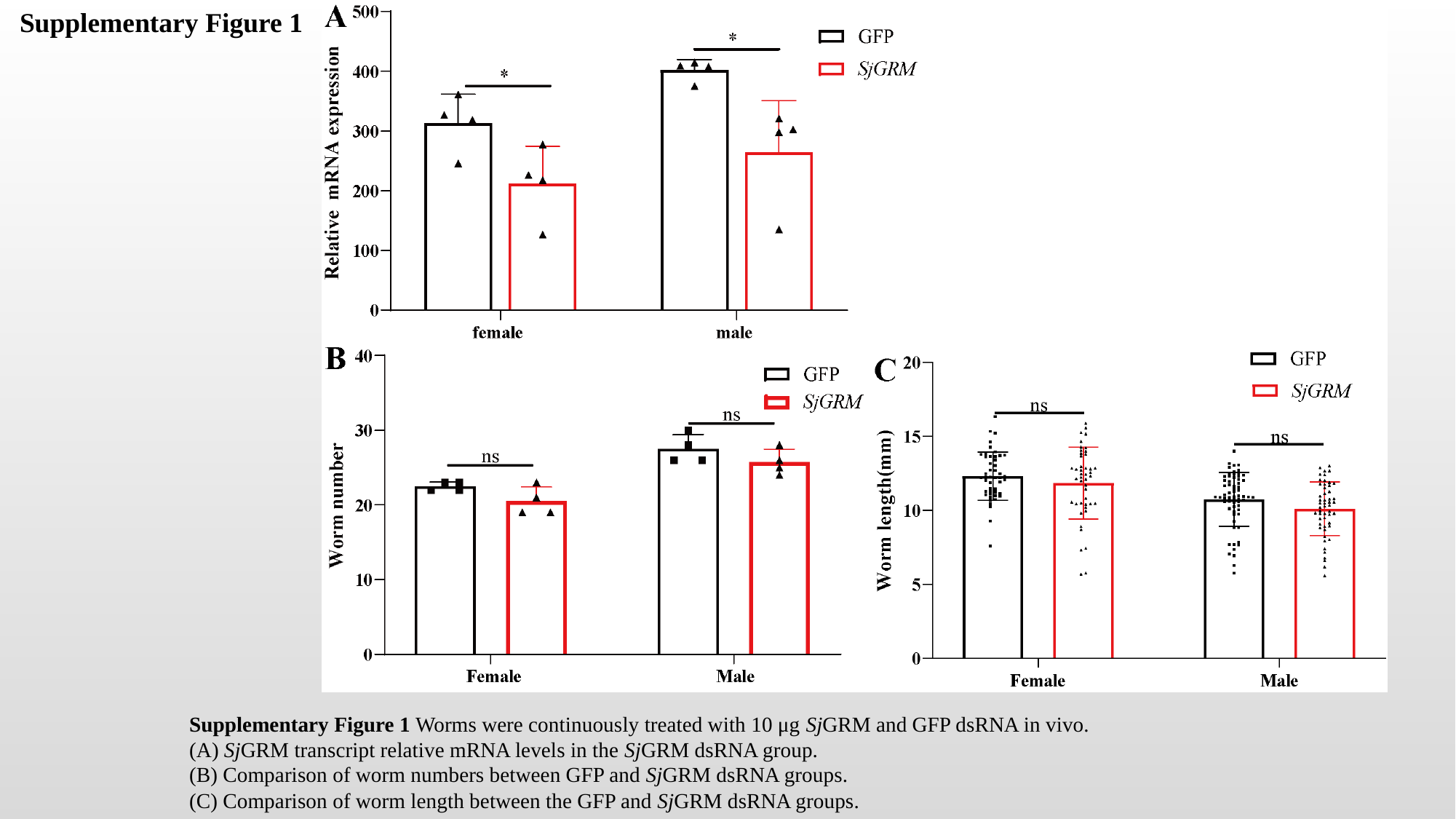

Supplementary Figure 1
Supplementary Figure 1 Worms were continuously treated with 10 μg SjGRM and GFP dsRNA in vivo.
(A) SjGRM transcript relative mRNA levels in the SjGRM dsRNA group.
(B) Comparison of worm numbers between GFP and SjGRM dsRNA groups.
(C) Comparison of worm length between the GFP and SjGRM dsRNA groups.

## Slide 2
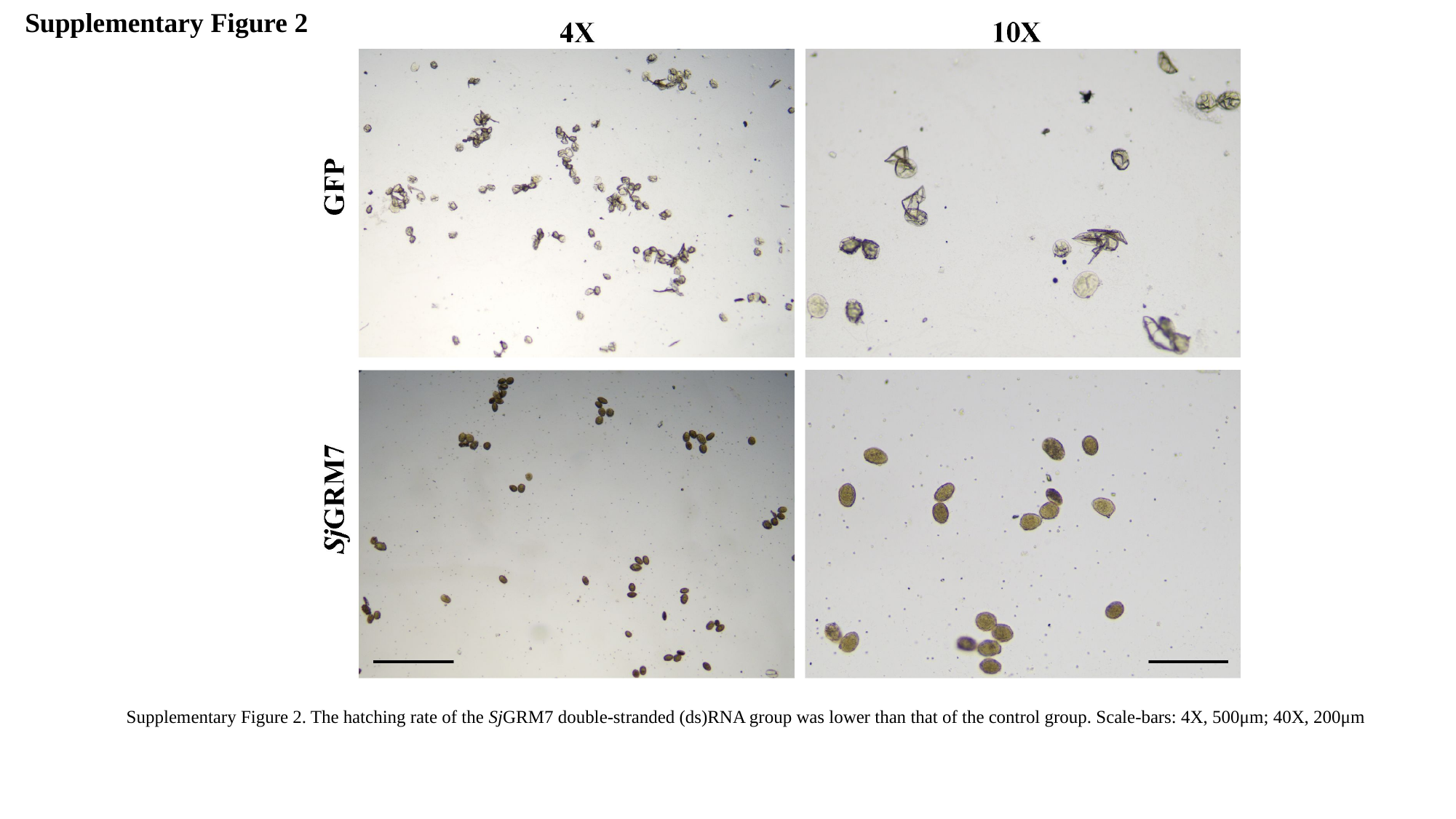

Supplementary Figure 2
Supplementary Figure 2. The hatching rate of the SjGRM7 double-stranded (ds)RNA group was lower than that of the control group. Scale-bars: 4X, 500μm; 40X, 200μm

## Slide 3
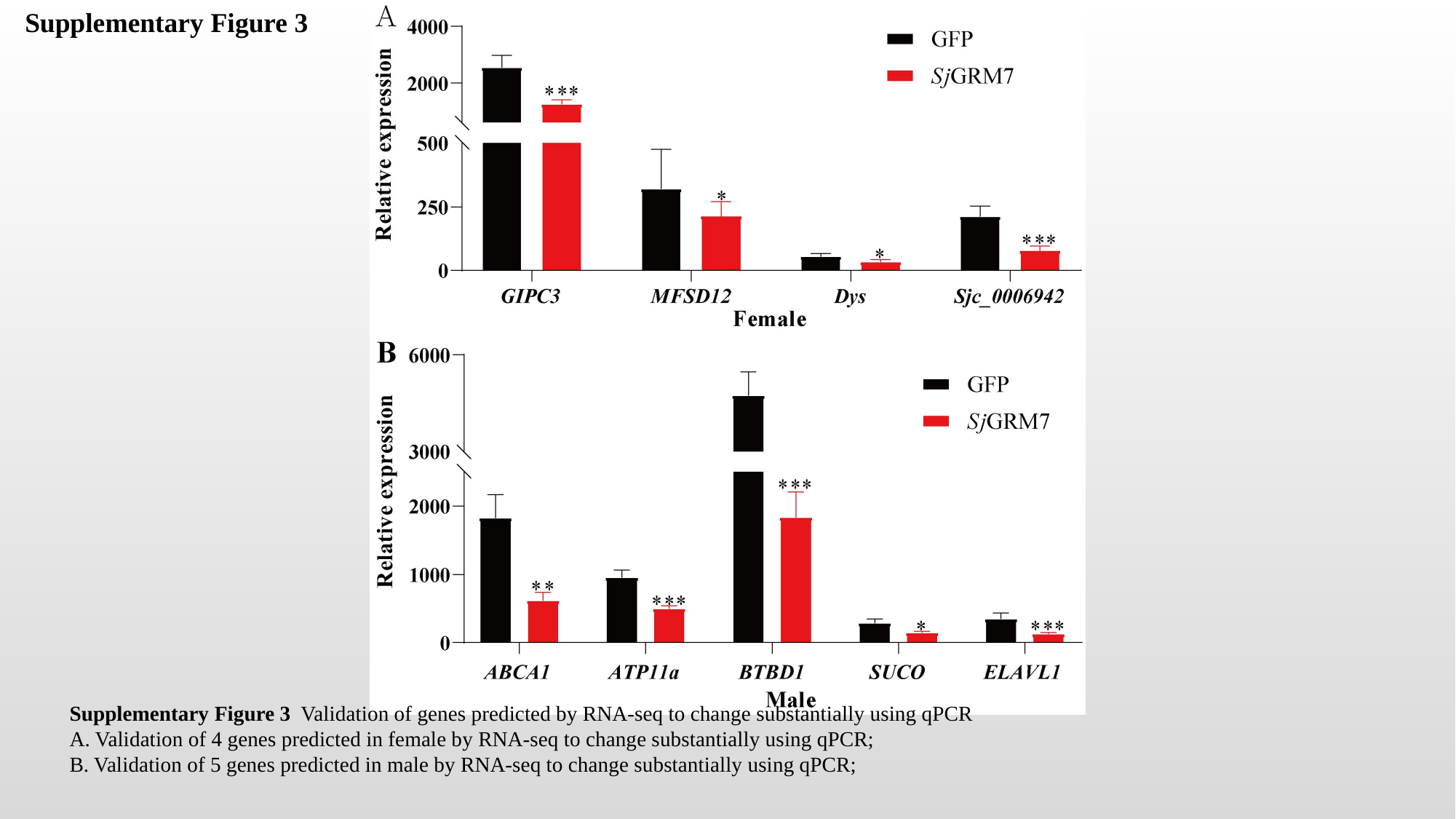

Supplementary Figure 3
Supplementary Figure 3 Validation of genes predicted by RNA-seq to change substantially using qPCR
A. Validation of 4 genes predicted in female by RNA-seq to change substantially using qPCR;
B. Validation of 5 genes predicted in male by RNA-seq to change substantially using qPCR;

## Slide 4
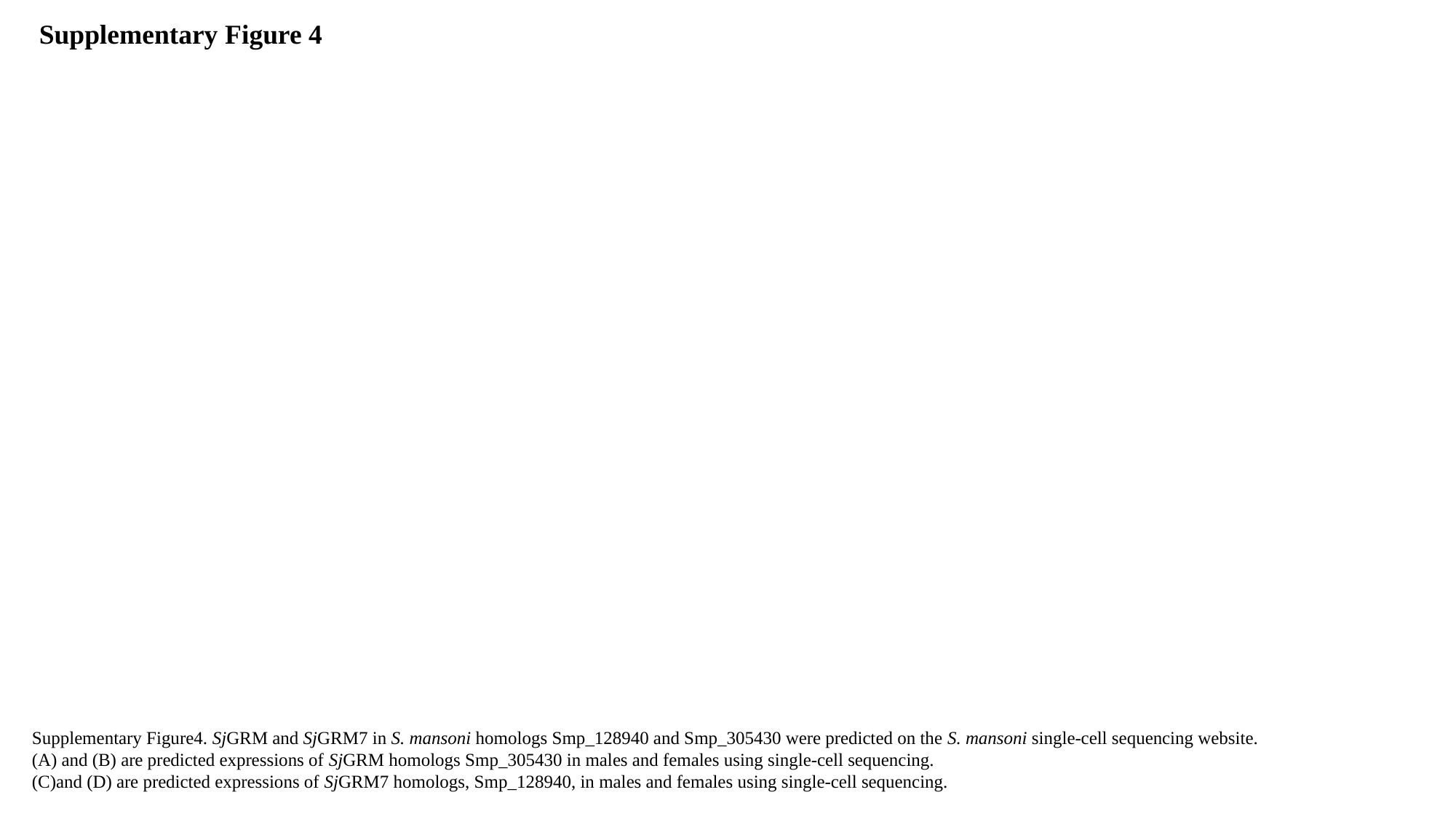

Supplementary Figure 4
Supplementary Figure4. SjGRM and SjGRM7 in S. mansoni homologs Smp_128940 and Smp_305430 were predicted on the S. mansoni single-cell sequencing website.
(A) and (B) are predicted expressions of SjGRM homologs Smp_305430 in males and females using single-cell sequencing.
(C)and (D) are predicted expressions of SjGRM7 homologs, Smp_128940, in males and females using single-cell sequencing.
